# National survey on the management of heart failure in individuals over 80 years of age in French geriatric care units

**DOI:** 10.1186/s12877-019-1215-y

**Published:** 2019-08-01

**Authors:** Clémence Boully, Jean-Sébastien Vidal, Etienne Guibert, Fanny Nisrin Ghazali, Alain Pesce, Bérengère Beauplet, Jean-Dominique Roger, Isabelle Carrière, Boubacar Timbely, Houria Idiri, Jean-Pierre Constensoux, Anne-Marie Durocher, Delphine Dubail, Marc Fargier, Claude Jeandel, Gilles Berrut, Olivier Hanon, on behalf of the SFGG study group, Etienne Guibert, Fanny Ghazali, Alain Pesce, Bérangère Beauplet, Jean-Dominique Roger, Isabelle Carrière, Boubacar Timbely, Houria Idiri, Jean-Pierre Constensoux, Anne-Marie Durocher, Delphine Dubail, Marc Fargier, Marie-Christine Godart, Marie-Agnès Manciaux, Angèle Néouze, Sabine Duranton Trevet, Régine Payen, Denata Mutelica, Oarda Bahri, Laurent Parrad, Aichouna Aroui-Sidelhadj, Adeline Lalu-Fraisse, Jean-Louis Supiot, Elise Thiel, Luc Walter, Guillaume Barthelat, Chantal Delemasure, Jean-Jacques Domerego, Catherine Maillet, Marcel Barjaud, Rachid Benaissa, Catherine Couturier, Marie-Line Gaubert Dahan, Alain Jean, Maurice Viala, Benoît Bartheleme, Edith Blanchet, Noël Blettner, Marie-Christine Bourdiol, Corinne Bruhat, Joanne Jenn, Eric Kiledjian, Sophie Lochet, Céline Martinand, Nicolas Ruillière, Hanitra Andrianasolo, Jean-Paul Cotinat, Aurélie Courrier, Laure de Decker, Denis Jamin, Bruno Martin, Odile Martinet, Samir Merbah, Elena Paillaud, Nathalie Ruel, Anne-Marie Barisic, Emmanuelle Comps, Marie Danet, Laurent de Bataille, Sandrine Estivin, Armelle Gentric, Dominique Huvent-Grelle, Léna Joffredo, Sophie Moulias, Agnès Rouaud, Dominique Adelving-Denuit, Emmanuel Alix, Marielle Berlioz-Thibal, Jean-Paul Bernard, Denis Branciforti, William Chatelier, Isabelle Defouilloy, Amandine Devun, Mourad Kacher, Marc Mennecart, Pascal Meyvaert, Patricia Rolland, Catherine Sagot, Philippe Veau, Marie-Thérèse Becerro Hallard, Souad Benhamed, Michel Béra, Frédéric Espagne, Virginie Fossey Diaz, Wiltord Jarzebowski, Camille Loiseau-Breton, Anne Sonnic, Marie-Mériadec Tanguy, Thalie Traissac, Valéry Antoine, Anne Chahwakilian, Denis Federico, Xavier Galimard, Hariniaina Jailany, Mireille Jurus-Frichet, Frederique Lenormand, Lise Lorentz, Jean-François Louvet, Annick Maltaverne, Valérie Revel Da Rocha, Sandrine Thibeaud, Sabiha Ahmine, Assia Bendris-Benaissa, Sophie Blanchemain, Clémence Boully, Cyrille Cantet, Cécile Charpentier, Pascal Chevalet, Dominique Clairet, Autila Crépin, Michel Davy, Jean-François Desson, Olivier Drunat, Yannick Gasnier, Yvette Giaccardi, Farid Hacini, Laëtitia Hengel-Di Nisi, Claire Houette, Claude Jeandel, Aurélie Lafargue, Eloïse Lefur-Musquer, Marina Monnier, Andriamasimanana Rakotoarisoa de Rozier, Claire Rouquet, Christel Salvietti, Alexia Serayet, Stephanie Vancompernolle, Pascale Vincent, Joelle Auffret Burguin, Edouard Beretti, Carine Bouayi, Yasmina Boudali, Hélène Chossonnery, Sébastien Colas, Marie-Hélène Fix, Isabelle Gantois, Anne-Laure Godard, Felipe Greslou, Stéphane Herbaud, Gabriel Malerba, Olivier Mellier, Eglantine Nemitz, Eric Pautas, Jean-Michel Pratico, Nicolas Redureau, Anne Richard, Dominique Rivière, Jean-Antoine Rosati, Aude Simon, Julien Zirnhelt, Cédric Annweiler, Marie-Ange Blanchon, Frédéric Bloch, Edouard Chaussade, Dominique Darmedru, Florence Delamarre-Damier, Sophie Deprecq, Michèle Escande, Catherine Hernandez, Mélanie Hervouet, Irina Ivancov, Florian Labourée, Carmelo Lafuente-Lafuente, Florence Leonel, Rachid Mahmoudi, Philippe Morlon, Etienne Ojardias, Lalaina Rakotoarisoa, Kokouvi Soadjede, Laurence Vaillard, Gabriel Abitbol, Sylvie Chaillou, Stéphanie Thomas, Marc Jegou, Anna Kearney-Schwartz, Véronique Larraillet, Gilles Loggia, Marie Lombard, Christelle Mischis, Brigitte Pichot Duclos, Saholy Razafindrainibe, Achille Tchalla, Catherine Terrat, Christine Yves Deville, Yassine Benamacht, Gilles Berrut, David Brugnon, Emmanuelle Ferry, Valery Gautier, Olivier Gilly, Emeline Proye, Georges Rakocevic, Typhaine Riaudel, Laure Schmitt, Patrick Friocourt, Jean-Sébastien Vidal, Olivier Hanon

**Affiliations:** 1 Assistance Publique des Hopitaux de Paris, Hopital Broca, 54-56 rue Pascal, 75013 Paris, France; 2 Sorbonne Paris-Cité, Université Paris-Descartes, Equipe d’Accueil 4468, Paris, France; 3 Ma Maison, Les Petites Sœurs des pauvres, 33000 Bordeaux, Paris France; 4 Ma Maison, Les Petites Sœurs des pauvres, 47000 Agen, Paris France; 5 Ma Maison, Les Petites Sœurs des pauvres, 17100 Saintes, Paris France; 6 GH Nord-Vienne, Pole 4, Gériatrie, Soins de suite, HAD, 86100 Chatellerault, Paris France; 7 CH Princesse-Grace, Centre Rainier III, 98000, Monaco, Monaco; 8 CHU de Caen, Départementfilière gériatrique, 14000 Caen, Paris France; 9 CH d’Annecy-Genevois, USLD La Tonnelle, 74600 Seynod, Paris France; 10 CH de Saint-Galmier, 42330 Saint-Galmier, Paris France; 11 CH de Meaux, Service soins de suite, 77100 Meaux, Paris France; 12 CHI Le Molinel, 59290 Wasquehal, Paris France; 13 EHPAD Mon Repos, 44140 Aigrefeuille-Sur-Maine, Paris France; 14 CHRU de Lille, Hôpital des Bateliers, 59037 Lille, Paris France; 15 ORPEA Clamart Maison Blanche, 92140 Clamart, France; 16 Orpea Résidence La Chanterelle, 93310 Le Pre-Saint-Gervais, Paris France; 17 CHU de Montpellier, Centre Antonin Balmès, 34000 Montpellier, Paris France; 18 CHU de Nantes, Hôpital Bellier, 44300 Nantes, Paris France

**Keywords:** Heart failure, Heart failure treatment, Age > 80 years old, Geriatric settings, Left ventricular ejection fraction

## Abstract

**Background:**

To evaluate the prevalence and management of heart failure (HF) in very old patients in geriatric settings.

**Methods:**

Members of the French Society of Geriatrics and Gerontology throughout France were invited to participate in a point prevalence survey and to include all patients ≥80 years old, hospitalized in geriatric settings, with HF (stable or decompensated) on June 18, 2012. General characteristics, presence of comorbidities, blood tests and medications were recorded.

**Results:**

Among 7,197 patients in geriatric institution, prevalence of HF was 20.5% (*n* = 1,478): (27% in acute care, 24.2% in rehabilitation care and 18% in nursing home). Mean age was 88.2 (SD = 5.2) and Charlson co morbidity score was high (8.49 (SD = 2.21)). Left ventricular ejection fraction (LVEF) was available in 770 (52%) patients: 536 (69.6%) had a preserved LVEF (≥ 50%), 120 (15.6%) a reduced LVEF (< 40%), and 114 (14.8%) a midrange LVEF (40–49%). Prescription of recommended HF drugs was low: 42.6% (629) used Angiotensin Converting Enzyme Inhibitors (ACEI) or Angiotensin Receptor Blockers (ARBs), 48.0% (709) β-blockers, and 21.9% (324) ACEI or ARB with β-blockers, even in reduced LVEF. In multivariate analysis ACEI or ARBs were more often used in patients with myocardial infarction (1.36 (1.04–1.78)), stroke (1.42 (1.06–1.91)), and diabetes (1.54 (1.14–2.06)). β blockers were more likely used in patients with myocardial infarction (2.06 (1.54–2.76)) and atrial fibrillation (1.70 (1.28–2.28)).

**Conclusion:**

In this large very old population, prevalence of HF was high. Recommended HF drugs were underused even in reduced LVEF. These results indicate that management of HF in geriatric settings can still be improved.

**Electronic supplementary material:**

The online version of this article (10.1186/s12877-019-1215-y) contains supplementary material, which is available to authorized users.

## Background

Low birth rates and higher life expectancy are transforming the demographics of Europe. The European Commission has estimated that by 2025**,** 20% of Europeans will be aged 65 years and older. The proportion of those aged 80 years and older in the Europe is expected to more than double between 2014 and 2050 from 5.1 to 10.9% [1]. Heart failure (HF) is a highly prevalent condition in the older population. After 80 years of age its prevalence varies between 15 and 20% [2–4]. Very old HF patients with multiple comorbidities are an important challenge for healthcare systems and are usually treated in geriatric institutions [5]. Despite a high prevalence and poor outcome, data on geriatric HF patients aged > 80 years from randomized controlled trials are limited and guidelines for this population are extrapolated from data on younger patients. The growing size of this population, however, requires more information on its optimal management. Although, HF treatment is thought to be beneficial to patients with reduced LVEF regardless of age, the existing literature indicates that the prescription of recommended treatments for HF is low in geriatric HF patients [6]. The aim of the current study was to carry out a point prevalence survey to provide up-to-date data on the prevalence and management of HF in individuals aged 80 years and older in geriatric settings in France and to explore factors influencing drug treatment.

## Methods

### Study design

This cross-sectional survey was initiated and conducted by the French Society of Geriatrics and Gerontology. A standardized questionnaire was emailed to 1600 geriatrician members of the French Society of Geriatrics and Gerontology throughout France, to evaluate the characteristics of patients with HF aged 80 years and older who were hospitalized in geriatric acute-care units (hospital department for acute patients with multiple chronic comorbidities), rehabilitation care units (hospital department for rehabilitation of patients with various neurological, orthopedic and other medical conditions following stabilization of their acute medical problems) or living in nursing homes (community-based institutions for disabled subjects with stable medical condition otherwise).

### Participants

Inclusion criteria were: age 80 years or older, diagnosis of decompensated or stable HF and hospitalized in a geriatric care unit for at least 24 h or living in a nursing home and present in the facility at 9 am on June 18, 2012.

Decompensated HF was defined as presence of acute symptoms or signs of HF according to ESC guidelines [7, 8]. Stable heart failure was defined as history of hospitalization for heart failure and unchanged HF symptoms and signs for at least 1 month [7].

### Data collection

For each patient, the following data were collected: demographic characteristics (age, gender, weight, systolic and diastolic blood pressure, heart rate) presence of cardiovascular comorbidities (hypertension, atrial fibrillation, history of myocardial infarction, history of stroke, diabetes, peripheral arterial disease, orthostatic hypotension) as well as non-cardiovascular comorbidities (anemia, renal insufficiency, malnutrition, dementia, falls (defined as ≥2 falls per year), chronic obstructive pulmonary disease (COPD), cancer), results of most recent blood tests (serum creatinine, electrolytes, albumin, hemoglobin, brain natriuretic peptide (BNP) and N-terminal brain natriuretic peptide (NTproBNP)), current medication (loop diuretic, thiazide diuretic, angiotensin converting enzyme inhibitor (ACEI), angiotensin II receptor blocker (ARB), β-blocker, aldosterone receptor antagonist, ivabradine, digoxin, nitrates), and whether the patients was advised to follow a low salt diet.

Antiplatelet and anticoagulant (therapeutic dose or prophylactic dose), and the total number of drug classes (for cardiovascular or non-cardiovascular drugs) were also recorder. Anemia was defined according to the World Health Organization criteria as a hemoglobin level lower than 13 g/dL in men and lower than 12 g/dL in women [9]. Estimated glomerular filtration rate (eGFR) was calculated using the Cockcroft and Gault formula [10]. Severe renal insufficiency was defined as eGFR < 30 ml/min. Malnutrition was defined as serum albumin level < 35 g/L.

The date of the last echocardiogram was noted as well as the left ventricular ejection fraction (LVEF) value. Finally, the burden of comorbidities was calculated using the age adjusted Charlson Comorbidity Index [11, 12].

### Procedure

Data were collected by the physician who was in charge of the patient on June 18, 2012. Physicians had 1 month until July 18, 2012 to send the data.

### Ethical consideration

The study was conducted in accordance with the ethical standards set forth in the Declaration of Helsinki (1983). The entire study protocol was approved by the ethics committee of Nantes (Groupe Nantais d’Ethique dans le Domaine de la Santé, France), and the study complied with the STROBE (Strengthening the Reporting of Observational Studies in Epidemiology) statement guidelines [13]. All patients’ data were anonymized in the local institution before they were uploaded to the central database.

### Data Analysis

First, demographic characteristics and clinical variables of the patients were analyzed in the whole sample and according to the type of geriatric care unit (acute care, rehabilitation care and nursing home) using descriptive statistics: means and standard deviations for continuous variables, and numbers and percentages for categorical variables. Comparisons were made with analysis of variance (ANOVA) for continuous data and with χ^2^ test for categorical data.

Because of the non-normal distribution of the brain natriuretic peptide and N-terminal brain natriuretic peptide variables, they were log-transformed for the calculation of the statistics but for the sake of clarity the non-logged figures are presented in the tables. Demographic characteristics and clinical variables of the patients were then compared between patients with decompensated and stable heart failure with Student’s *t*-tests for the continuous variables and χ^2^ tests for the categorical variables.

Second, the differences in treatment between 3 groups of LVEF (preserved LVEF (≥ 50%), midrange LVEF (40–49%) and reduced LVEF (< 40%) [7] were graphically presented in a barplot and compared with χ^2^ tests.

Third, comparisons were made between patients with and without ACEI or ARBs treatment and between patients with and without β-blocker treatment. Two logistic regression models were built, one with ACEI or ARB and one with β-blockers as dependent variable and factors univariately associated (*p* < 0.10) with the dependent variables. In these 2 models, LVEF was not included in the dependent variables because it was only available in 52% of the sample. The results were graphically presented on two forest plots. Because ACEI, ARB and β-blockers are medication recommended for HF with reduced EF, as sensitivity analysis, we built 2 other models, one with ACEI or ARB and one with β-blockers as dependent variables restricted to patients with reduced LVEF.

All analyses were two-sided and a *p*-value < 0.05 was considered statistically significant. Data analysis was performed using R software version 3.2.3, (R Core Team (2014). R: A language and environment for statistical computing. R Foundation for Statistical Computing, Vienna, Austria. URL http://www.R-project.org/).

## Results

### Participants and patient characteristics

A total of 183 practitioners from 134 geriatric institutions participated in the survey: 58 from acute care units, 50 from rehabilitation care units and 75 from nursing homes. These practitioners were in charge of 7,197 patients: 1,397 acute care, 1,331 rehabilitation care and 4,469 nursing homes. Out of these 7,197 patients, 1,478 presented with HF on the day of the survey and were included in the study giving an overall HF prevalence of 20.5% (95% confidence interval 19.8–21.3%), 27.1% (25.2–29.1%) (*N* = 379) in acute care units, 24.2% (22.3–26.2%) (*N* = 322) in rehabilitation care units and 17.6% (16.7–18.5%) (*N* = 774) in nursing homes.

Demographic characteristics and past medical history of the sample are shown Table 1. The mean (SD) age was 88.2 (SD = 5.2) years and 68.9% (*N* = 1014) were women. The mean age adjusted Charlson comorbidity index score was 8.49 (2.21). The most common comorbidities were hypertension (77.6%), malnutrition (64.1%), anemia (59.9%), dementia (52.3%), atrial fibrillation (43.2%), depression (34.1%), history of myocardial infarction (28.5%), COPD (24.6%), diabetes (22.1%), peripheral arterial disease (20.0%), history of stroke (19.8%), orthostatic hypotension (18.5%) and cancer (16.3%). Patients were taking on an average of 8.43 (3.29) different drugs. Few patients (3.8% (57)) had a very low systolic blood pressure (< 100 mmHg in a sitting position). Heart rate was greater than 70 beats per minute (bpm) in 56.4% (816) of patients. In patients with permanent atrial fibrillation on the electrocardiogram, 40.7% (247) had a heart rate lower than 70 bpm whereas in patients in sinus rhythm on the electrocardiogram, 46.5% (369) had a heart rate lower than 70 (*p* = 0.04).

**Table 1:** Baseline characteristics in the whole sample and according to the care settings

General characteristics, % (N)	Whole sample	Acute care	Rehabilitation care	Nursing home	p^a^
	*N* = 1478	*N* = 382	N = 322	N = 774	
Heart failure type					
Decompensated	21.4 (316)	51.2 (195)	16.6 (53)	8.79 (68)	<.0001
Stable	78.6 (1158)	48.8 (186)	83.4 (266)	91.2 (706)	
Age (years), M (SD)	88.2 (5.2)	87.8 (4.9)	87.3 (5.2)	88.7 (5.3)	<.0001
Women	68.9 (1014)	61.4 (234)	61.4 (197)	75.7 (583)	<.0001
Weight (Kg), M (SD)	64.6 (15.4)	66.8 (16.7)	63.4 (15.4)	64.1 (14.7)	0.005
Systolic blood pressure (mmHg), M (SD)	126 (19)	126 (20)	124 (19)	127 (18)	0.04
Diastolic blood pressure (mmHg), M (SD)	68.9 (11.5)	68.7 (12.3)	66.9 (10.8)	69.9 (11.4)	0.0005
Orthostatic hypotension	18.5 (137)	15.5 (28)	16.7 (26)	20.6 (83)	0.26
Heart rate (beats per minute), M (SD)	74.0 (12.7)	76.3 (13.8)	74.9 (13.0)	72.4 (11.7)	<.0001
Diabetes mellitus	22.1 (326)	23.0 (88)	25.2 (81)	20.3 (157)	0.18
Hypertension	77.6 (1144)	76.4 (291)	76.9 (594)	80.4 (259)	0.37
History of stroke	19.8 (293)	20.4 (78)	17.7 (57)	20.4 (158)	0.55
History of myocardial infarction	28.5 (421)	32.8 (125)	37.2 (119)	22.9 (177)	<.0001
Peripheral arterial disease	20.0 (295)	20.2 (77)	19.6 (152)	20.6 (66)	0.93
Acute infection	44.1 (436)	46.9 (149)	42.6 (106)	43.0 (181)	0.49
Dementia	52.3 (772)	39.0 (149)	46.0 (148)	61.4 (475)	<.0001
Depression	34.1 (503)	22.8 (87)	32.7 (105)	40.2 (311)	<.0001
History of falls	32.5 (480)	33.0 (126)	44.1 (142)	27.4 (212)	<.0001
COPD	24.6 (363)	24.9 (95)	22.4 (72)	25.3 (196)	0.58
Cancer	16.3 (241)	16.2 (62)	18.9 (61)	15.2 (118)	0.32
eGFR (mL/min), M (SD)	35.1 (16.3)	33.7 (15.7)	35.5 (16.2)	35.6 (17.5)	0.19
Hemoglobin (g/dL), M (SD)	11.8 (1.7)	11.6 (1.8)	11.5 (1.5)	12.1 (1.6)	<.0001
Anemia	59.9 (864)	63.5 (240)	53.7 (400)	70.2 (224)	<.0001
Albumin (g/L), M (SD)	32.7 (5.2)	32.1 (5.7)	31.6 (5.1)	33.8 (4.8)	<.0001
Malnutrition (albumin < 35 g/l)	64.1 (754)	65.9 (220)	60.1 (336)	70.0 (198)	0.01
Brain natriuretic peptide (pg/mL), M (SD)	993 (1793)	1329 (2282)	1208 (1858)	622 (1160)	0.004
NTproBNP (pg/mL), M (SD)	5926 (9587)	8489 (12237)	7165 (9517)	3317 (6000)	<.0001
LVEF %, M (SD)	52.9 (14.4)	51.3 (15.6)	50.0 (14.4)	55.7 (13.1)	<.0001
LVEF level in 3 classes					
LVEF < 40%	15.6 (120)	18.8 (41)	22.1 (44)	9.92 (35)	
LVEF 40–49%	14.8 (114)	13.8 (30)	16.6 (33)	14.4 (51)	0.0009
LVEF ≥50%	69.6 (536)	67.4 (147)	61.3 (122)	75.6 (267)	
Atrial fibrillation	43.2 (614)	48.0 (181)	45.1 (142)	39.9 (291)	0.03
Antiplatelet drugs	44.4 (655)	43.0 (163)	46.6 (150)	44.2 (342)	0.63
Statins	21.4 (316)	23.9 (91)	27.4 (88)	17.7 (137)	0.0007
Anticoagulants	47.0 (694)	63.9 (244)	60.2 (194)	33.1 (256)	<.0001
Total number of drugs, M (SD)	8.43 (3.29)	8.30 (3.43)	8.71 (3.40)	8.37 (3.17)	0.22
Age-adjusted Charlson score, M (SD)	8.49 (2.21)	8.48 (2.27)	8.42 (1.99)	8.66 (2.60)	0.25

*% (N)* percentage (number); *M (SD)* mean (standard deviation), *eGFR* estimated glomerular filtration rate calculated with Cockcroft formula, *LVEF* left ventricular ejection fraction, *COPD* chronic obstructive pulmonary disease

^a^ ANOVA test or χ^2^

Only 780 (52.8%) patients had an available echocardiogram in their medical records. Ninety (11.6%) had an echocardiogram in the past week, 135 (17.4%) in the past 7–30 days, 181 (23.4%) in the past 1–6 months, 89 (11.5%) in the past 6–12 months, 107 (13.8%) in the past 12–24 months, and 172 (22.2%) over 2 years. Among the 780 patients with an available echocardiogram 770 had an LVEF calculated. The mean LVEF was 52.9% (14.4). 69.6% percent (536) had a preserved LVEF (LVEF ≥50%), 15.6% (120) had a reduced LVEF (LVEF < 40%), and 14.8% (114) had a midrange ejection fraction LVEF (40–49%).

Decompensated HF was reported in 21.4% (*n* = 316) and stable HF in 78.4% (*n* = 1158) for 4 subjects the information was missing.

For decompensated HF, the main precipitating factors leading to decompensation were infections (44.1%), atrial fibrillation (29.9%), anemia (19.7%) and acute coronary syndrome (10.0%).

Overall level of natriuretic peptide was elevated in this population. NTproBNP and BNP were higher in the decompensated than in the stable HF patients (10407 pg/mL (12393) vs. 4208 pg/mL (7621), *p* < .0001 and 1527 pg/mL (2141) vs. 729 pg/mL (15311), p < .0001).

### Heart failure drug treatments

The type of HF drug treatments taken by patients is shown in Table 2. Patients with decompensated HF were more likely to receive loop diuretics and nitrates whereas patients with stable HF had higher prescriptions of ACEI or ARBs either alone or in combination with a β-blocker. A low salt diet was advised in 9.76% (144) most often in patients with decompensated HF (16.5% (52) vs. 7.95% (92), *p* < 0.0001). Overall, prescription of recommended HF drug treatments was low whether patients had decompensated or stable HF: 629 (42.6%) received ACEI or ARBs and 709 (48.0%) received β-blockers. The prescription of an ACEI or ARB in combination with a β-blocker was also very low (21.9% (324)), as was the prescription of aldosterone antagonists (5.95% (88)), ivabradine (0.88% (13)), nitrates (13.5% (199)) and digoxin (8.32% (123)).

**Table 2:** Therapy in the whole sample and according to decompensated and stable heart failure

Characteristics, % (N)	Whole sample	Decompensated HF	Stable HF	p^a^	P^b^
	N = 1478	N = 316	N = 1158		
Low salt diet	9.76 (144)	16.5 (52)	7.95 (92)	<.0001	<.0001
Loop diuretic	64.7 (957)	86.7 (274)	58.9 (682)	<.0001	<.0001
Thiazide diuretic	5.68 (84)	5.06 (16)	5.87 (68)	0.68	0.63
Angiotensin converting enzyme inhibitor (ACEI)	31.7 (468)	27.8 (88)	32.7 (379)	0.11	0.12
Angiotensin receptor blocker (ARB)	11.2 (165)	7.91 (25)	12.1 (140)	0.05	0.03
ACEI or ARB	42.6 (629)	35.4 (112)	44.6 (516)	0.004	0.005
β-blocker (BB)	48.0 (709)	48.4 (153)	47.8 (553)	0.89	0.71
BB and (ACEI or ARB)	21.9 (324)	16.8 (53)	23.4 (271)	0.01	0.02
Aldosterone antagonists	5.95 (88)	5.38 (17)	6.13 (71)	0.71	0.69
Ivabradine	0.880 (13)	1.27 (4)	0.777 (9)	0.63	0.43
Digoxin	8.32 (123)	8.23 (26)	8.38 (97)	0.99	0.96
Nitrates	13.5 (199)	17.1 (54)	12.5 (145)	0.04	0.04
Antiplatelet	44.3 (652)	38.5 (121)	45.9 (531)	0.02	0.02
Statins	21.4 (316)	18.2 (57)	22.4 (259)	0.12	0.14
Anticoagulants	47.0 (694)	64.6 (204)	42.2 (489)	<.0001	<.0001
Therapeutic anticoagulants	65.6 (453)	68.3 (138)	64.5 (315)	0.39	0.25
Anticoagulant prophylaxis	34.4 (238)	31.7 (64)	35.5 (173)		
Total number of drugs	8.43 (3.29)	8.55 (3.41)	8.39 (3.26)	0.46	0.35

^a^ ANOVA test or χ^2^

^b^ Logistic regression adjusted for age and sex

### Distribution of drug treatments according to preserved midrange and reduced LVEF

In the 770 patients with an available LVEF, there was low use of recommended HF drug treatments in patients with reduced LVEF. 64 (53.3%) used ACEI or ARB, 82 (68.3%) used β-blocker and 50 (41.7%) used a combination of β-blocker with an ACEI or ARB.

**Fig. 1:**
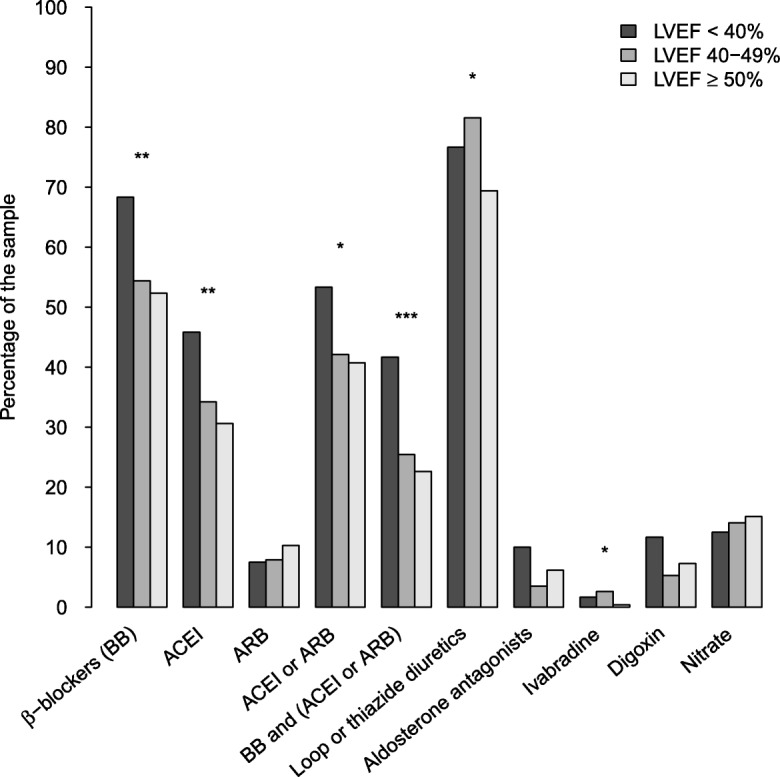
Distribution of treatments according to LVEF level. Overall difference between the 3 groups, * *p* < 0.05; ** *p* < 0.01; *** *p* < 0.001. Diuretics, Loop and thiazide diuretics; ACEI, Angiotensin converting enzyme inhibitor; ARB, Angiotensin receptor blocker

However, the use of ACEI or ARB or β-blocker was higher in patients with reduced LVEF than in patients with preserved or midrange LVEF (See Fig. 1). There was also more prescription of loop or thiazide diuretics and ivabradine among those with reduced or midrange LVEF than among those with preserved LVEF. No difference was observed for aldosterone antagonists, digoxin and nitrates according to LVEF categories.

### Factors associated with prescription of ACEI or ARB in very old patients with HF

Additional file 1: Table S1 shows the characteristics of patients according to ACEI or ARB use.

**Fig. 2:**
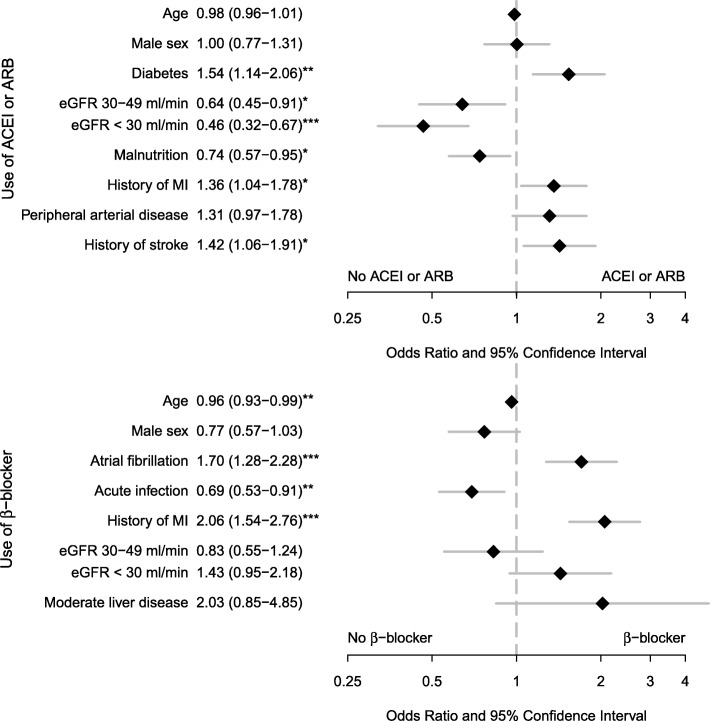
Factors associated with ACE inhibitor or ARB and with β-blocker prescription. * *p* < 0.05; ** *p* < 0.01; *** *p* < 0.001. ACEI, Angiotensin converting enzyme inhibitor; ARB, Angiotensin receptor blocker; eGFR, estimated glomerular filtration rate calculated with Cockcroft formula; MI, myocardial infarction

In a multivariate analysis, after adjustment for predictors of ACEI or ARB prescription identified by univariate analysis, ACEI or ARBs were more likely prescribed in patients with cardiovascular diseases including history of myocardial infarction (OR: 1.36 (95% CI: 1.04–1.78)), history of stroke (OR: 1.42 (95% CI: 1.06–1.91)), and diabetes (OR: 1.54 (95% CI: 1.14–2.06)) (Fig. 2). Determinants of the non-prescription of ACEI or ARB included malnutrition (serum albumin levels < 35 g/L) (OR: 0.74 (95% CI: 0.57–0.95)) and eGFR between 30 and 50 ml/min (OR: 0.64 (95% CI: 0.45–0.91)) and eGFR < 30 ml/min (OR: 0.46 (95% CI: 0.32–0.67)).

When the model was run among patients with reduced LVEF, predictors of ACEI or ARB prescription were then diabetes (OR: 2.23 (95% CI: 1.18–4.31), *p* = 0.02), history of myocardial infarction (OR: 3.26 (95% CI: 1.83–5.96), *p* < .0001) and eGFR between 30 and 50 ml/min (OR: 0.22 (95% CI: 0.09–0.54), *p* = 0.001) and eGFR < 30 ml/min (OR: 0.35 (95% CI: 0.14–0.83), *p* = 0.02).

### Factors associated with prescription of β-blockers in very old patients with HF

Additional file 1: Table S2 shows the characteristics of patients according to β-blockers use.

Multivariate analysis indicated that β blockers were more likely prescribed in patients with myocardial infarction (OR: 2.06 (95% CI: 1.54–2.76)) and atrial fibrillation (OR: 1.70 (95% CI: 1.28–2.28)) (Fig. 2). Determinants of the non-prescription of a β-blocker were older age (OR: 0.96 (95% CI: 0.93–0.99) and infection (OR: 0.69 (95% CI: 0.53–0.91)).

When the model was run among patients with reduced LVEF, β blockers prescription was then no more associated with any of the predictors. However myocardial infarction (OR: 1.77 (95% CI: 0.98–3.24), *p* = 0.06), atrial fibrillation (OR: 1.68 (95% CI: 0.94–3.06), *p* = 0.08) and infection (OR: 0.61 (95% CI: 0.35–1.08), *p* = 0.09) were marginally associated with β blocker prescriptions.

## Discussion

This large cross-sectional survey study provides information on the current management of very old patients with HF in geriatric settings in France. The overall prevalence of HF was 20.5% (ranging from 27% in acute care departments to 18% in nursing home).

Prescriptions of guideline-recommended HF drug treatments were low whether patients had decompensated or stable HF. Less than half of the subjects received ACEI or ARBs or β-blockers. The combination of an ACEI or ARB with a β-blocker was prescribed in less than 22% of the subjects.

The prevalence of HF in this study is in the same range as reported in a systematic review which indicates that about 20% (15–45%) of nursing homes residents are affected by HF [4].

Published studies on the management of HF in geriatric patients with multiple comorbidities are sparse, even though this population is growing rapidly. In the current study, geriatric HF patients had a high number of comorbidities, in particular non-cardiovascular such as malnutrition, dementia and falls that are generally not evaluated in clinical trials. This result is in line with the SAGE study indicating that in nursing home 32% of HF patients have at least six other concurrent diseases [14] and with the Swedish Heart Failure Registry database, that reported a higher incidence of cardiovascular and non-cardiovascular comorbidities in patients 85 years and older [15].

In the current study, only 53% of patients had an available echocardiogram in their medical records, and among those, 70% had a preserved LVEF. These data highlight the difficulties in obtaining an echocardiogram for patients in geriatric care setting because of the difficulties in transporting patients to a cardiology unit, and lower physician demand for diagnostic confirmation.

Our results confirm previous observations of a higher prevalence of HF with preserved LVEF among the very old [2, 16–19]. In octogenarians hospitalized for HF in the Euro Heart Failure Survey (EHFS), only 38.4% of patients had a known LVEF and in 60% of these LVEF were preserved [16].

In our study, regardless of whether HF was stable or decompensated, the prescription of recommended treatments for HF such as ACEI (or ARB) and β-blocker was low. Particularly the combination of an ACEI (or ARB) and a β-blocker was very low (less than a quarter of subjects). Among subjects with reduced LVEF, the rate of prescriptions of ACEI (or ARB) or β-blocker was still sub-optimal (53% for ACEI or ARB and 68% % for β-blocker) even though they were more often prescribed than in midrange and preserved LVEF. The greater prevalence of HF with preserved LVEF was therefore not the only reason for the low use of ACEI (or ARB) and β-blockers in older patients. The underuse of recommended HF treatments in the very old age has already been reported in other studies [6, 15, 16, 20–22]. In a study collecting data on 19 long-term care facilities only 41 and 38% prescription of ACEI and β-blockers respectively were reported in eligible HF patients [6]. In a large Canadian study in older home-care patients only 28% were receiving the recommended combination therapy of an ACEI and β-blocker [20]. Similar findings were reported in the Swedish Heart Failure Registry database [15] and in an Italian cardiology database [22]. A comparison of HF therapy between the Euro Heart Failure survey I and the EHFS II found a significant increase in prescription rates of recommended HF drugs in HF octogenarians at discharge in EHFS II [2], but these data mainly concerned patients hospitalized in cardiology units. Our data indicate that prescription guidelines remain less implemented in geriatric settings. A number of potential reasons for the low prescription of recommended therapies in very old HF patients can be hypothesized. Some factors may be related to the patients (comorbidities, poor tolerance, non-adherence) and some to the prescribers (fear of side effects, lack of awareness of guidelines, diagnostic uncertainty, focus on symptomatic improvement rather than outcome, reluctance to modify existing therapies in very old patients) [16, 23]. Moreover, the lack of definite evidence from specific randomized trial in very old population may also explain the low use of ACEI or beta-blocker. However, all the randomized trials conducted in HF patients with reduced LVEF have shown a benefit of those drugs regardless of age. Also, there is evidence from small trials and observational datasets that HF medications do improve outcomes in older HF patients, including quality of life related outcomes [2, 16].

Finally, the difficulty to get cardiologic advice in a nursing home may also explain the lower prescription of recommended HF drugs in this population, suggesting a potential interest of telemedicine in this population. Multimodal and guideline-based HF management may improve HF patients in geriatrics settings [24]. Specific education on HF for geriatricians and the development of specific tools like portable ultrasound scanner should be investigated in geriatric care units or nursing homes [24].

Similar to previous studies [16], there was a high use of diuretics reflecting the importance of reducing symptoms and maintaining quality of life as the main goals of treatment in very old HF patients. The low use of digoxin was in line with guidelines that caution against their use in the elderly and in patients with reduced renal function [7]. We observed a low use of nitrates in decompensated HF probably because of an increased risk of orthostatic hypotension in the elderly.

Our data show that in very old HF patients managed in geriatric care units or nursing homes, the factors determining the prescription of first-line HF treatments (ACEI or ARB and β-blocker) were history of myocardial infarction and stroke, diabetes for ACEI or ARBs and history of myocardial infarction and atrial fibrillation for β-blockers. Factors influencing the nonprescription of HF treatment were non-cardiovascular comorbidities (malnutrition and renal insufficiency for ACEI (or ARBs) and infections for β-blockers).

Among patients with reduced LVEF, history of stroke and malnutrition were not any more significantly associated with prescription of ACEI or ARBs. For prescription of β-blockers, history of myocardial infarction and atrial fibrillation and infections were not any more significant but the estimates were in the same range as in the whole sample. This is in all likelihood related to a lack of power due to the small sample size.

Over half of the patients in the current study had on average a heart rate faster than 70 bpm, even though a number of large-scale studies have shown that elevated heart rate is associated with morbidity and mortality in HF patients with reduced or preserved LVEF independently of age [25–29].

The OPTIMIZE-HF Registry observed that among 10,696 patients with HF and LVEF < 40, 28.6% did not receive β-blockers and only 6.7% of patients received the target dose of β-blockers [30]. These results stress the difficulties of obtaining such goals in real life mainly in the older people.

Interestingly, BNP or NTproBNP levels remained very high even in stable HF, suggesting a suboptimal treatment. Meanwhile, natriuretic peptide level increases with age and comorbidities (renal dysfunction, atrial fibrillation, left ventricular hypertrophy…) making it difficult to interpret in geriatric population [31].

Our study has several limitations. It is an observational study performed on 1 day and only comprising hospitalized or institutionalized older individuals who might not be representative of the whole elderly population. A reporting bias cannot be ruled out as the accuracy and completeness of the data were entirely reliant upon physicians’ declarations, although the questionnaire was designed to limit variability in readers’ interpretations by asking only factual data. In addition, while selection bias could not be eliminated, investigators were asked to take into account all the patients in their care on the day of the survey. Information on duration and dose of treatments was not collected. No follow-up was available to analyze the adequacy between underuse of recommended HF and mortality. Activities of daily living and frailty status were not recorded in this study because it was difficult to assess in hospitalized patient with HF and unfortunately their status before the hospitalization had not been assessed. Thus, this potential cause for the underuse of medications was not assessed. Meanwhile cognitive status that is a classical cause of underuse of medications was taken into account. Lastly data were collected in 2012 and might not exactly reflect the current status.

The study also had some strengths. This study provides important data on the management of HF failure in patients over 80 years of age in geriatric care unit or nursing home. Few data exist for this population, particularly in real-life settings. Our study had a large sample size and included very old (mean age 88.2 (5.2)) non-selected ‘real-life’ HF patients with numerous comorbidities like dementia, malnutrition, anemia, renal insufficiency, depression, orthostatic hypotension (Charlson score = 8.49). Lastly few studies have analyzed the management of HF according to 3 classes of LVEF (reduced, midrange and preserved LVEF) in geriatric patients.

## Conclusion

Our results show a high prevalence of HF in patients aged 80 years and older cared for in a geriatric care unit or nursing home (20.5%), characterized by a high prevalence of preserved LVEF and a high burden of cardiovascular as well as non-cardiovascular comorbidities. There was a low prescription of recommended treatment in the overall HF population with less than half of patients receiving an ACEI or ARB and less than a quarter receiving an ACEI or ARB in combination with a β-blocker. This under-prescription was also observed in patients with reduced LVEF. Overall, these results indicate that management of HF in patients cared for in geriatric settings can still be improved.

## Additional file

Additional file 1 **Table S1** Characteristics of patients according to ACEI or ARB use. **Table S2** Characteristics of patients according to β-blocker use. (DOCX 47 kb)

## Data Availability

The datasets used and/or analyzed during the current study are available from the corresponding author on reasonable request.

## Abbreviations

ACEI: Angiotensin converting enzyme inhibitor
ANOVA: Analysis of variance
ARB: Angiotensin II receptor blocker
BNP: Brain natriuretic peptide
COPD: Chronic obstructive pulmonary disease
EHFS: Euro heart failure survey
HF: Heart failure
LVEF: Left ventricular ejection fraction
NTproBNP: N-terminal brain natriuretic peptide
SD: Standard deviation
STROBE: Strengthening the reporting of observational studies in epidemiology

## References

[1] Eurostat. People in the EU – population projections. 2015. https://ec.europa.eu/eurostat/statistics-explained/index.php/People_in_the_EU_-_population_projections#Population_projections.

[2] Komajda M, Hanon O, Hochadel M, Lopez-Sendon JL, Follath F, Ponikowski P (2009). Contemporary management of octogenarians hospitalized for heart failure in Europe: euro heart failure survey II. Eur Heart J.

[3] Paren P, Schaufelberger M, Bjorck L, Lappas G, Fu M, Rosengren A (2014). Trends in prevalence from 1990 to 2007 of patients hospitalized with heart failure in Sweden. Eur J Heart Fail.

[4] Daamen MAMJ, Hamers JPH, Gorgels APM, Brunner-La Rocca H, Tan FES, van Dieijen-Visser MP (2015). Heart failure in nursing home residents; a cross-sectional study to determine the prevalence and clinical characteristics. BMC Geriatr.

[5] Laveau F, Hammoudi N, Berthelot E, Belmin J, Assayag P, Cohen A (2017). Patient journey in decompensated heart failure: an analysis in departments of cardiology and geriatrics in the greater Paris University hospitals. Arch Cardiovasc Dis.

[6] Mann JL, Evans TS (2006). A review of the management of heart failure in long-term care residents. Consult Pharm.

[7] Ponikowski P, Voors AA, Anker SD, Bueno H, Cleland JGF, Coats AJS (2016). 2016 ESC guidelines for the diagnosis and treatment of acute and chronic heart failure: the task force for the diagnosis and treatment of acute and chronic heart failure of the European Society of Cardiology (ESC)developed with the special contribution of the heart failure association (HFA) of the ESC. Eur Heart J.

[8] McMurray JJV, Adamopoulos S, Anker SD, Auricchio A, Bohm M, Dickstein K (2012). ESC guidelines for the diagnosis and treatment of acute and chronic heart failure 2012: the task force for the diagnosis and treatment of acute and chronic heart failure 2012 of the European Society of Cardiology. Developed in collaboration with the heart failure association (HFA) of the ESC. Eur Heart J.

[9] WHO (1968). Nutritional anaemias. Report of a WHO scientific group. World Health Organ Tech Rep Ser.

[10] Cockcroft DW, Gault MH (1976). Prediction of creatinine clearance from serum creatinine. Nephron.

[11] Charlson ME, Pompei P, Ales KL, MacKenzie CR (1987). A new method of classifying prognostic comorbidity in longitudinal studies: development and validation. J Chronic Dis.

[12] Charlson M, Szatrowski TP, Peterson J, Gold J (1994). Validation of a combined comorbidity index. J Clin Epidemiol.

[13] Rouaud A, Hanon O, Boureau A, Chapelet G, de Decker L (2015). Comorbidities against quality control of VKA therapy in non-valvular atrial fibrillation: a French national cross-sectional study. PLoS One.

[14] Gambassi G, Forman DE, Lapane KL, Mor V, Sgadari A, Lipsitz LA (2000). Management of heart failure among very old persons living in long-term care: has the voice of trials spread? The SAGE study group. Am Heart J.

[15] Holmstrom A, Sigurjonsdottir R, Edner M, Jonsson A, Dahlstrom U, Fu ML (2013). Increased comorbidities in heart failure patients >/= 85 years but declined from >90 years: data from the Swedish heart failure registry. Int J Cardiol.

[16] Komajda M, Hanon O, Hochadel M, Follath F, Swedberg K, Gitt A (2007). Management of octogenarians hospitalized for heart failure in euro heart failure survey I. Eur Heart J.

[17] Owan TE, Hodge DO, Herges RM, Jacobsen SJ, Roger VL, Redfield MM (2006). Trends in prevalence and outcome of heart failure with preserved ejection fraction. N Engl J Med.

[18] Kitzman DW, Gardin JM, Gottdiener JS, Arnold A, Boineau R, Aurigemma G (2001). Importance of heart failure with preserved systolic function in patients > or = 65 years of age. CHS research group. Cardiovascular health study. Am J Cardiol.

[19] McMurray JJV, Carson PE, Komajda M, McKelvie R, Zile MR, Ptaszynska A (2008). Heart failure with preserved ejection fraction: clinical characteristics of 4133 patients enrolled in the I-PRESERVE trial. Eur J Heart Fail.

[20] Foebel AD, Heckman GA, Hirdes JP, Tyas SL, Tjam EY, McKelvie RS (2011). Clinical, demographic and functional characteristics associated with pharmacotherapy for heart failure in older home care clients: a retrospective, population-level, cross-sectional study. Drugs Aging.

[21] Mahjoub H, Rusinaru D, Souliere V, Durier C, Peltier M, Tribouilloy C (2008). Long-term survival in patients older than 80 years hospitalised for heart failure. A 5-year prospective study. Eur J Heart Fail.

[22] Pulignano G, Del Sindaco D, Tavazzi L, Lucci D, Gorini M, Leggio F (2002). Clinical features and outcomes of elderly outpatients with heart failure followed up in hospital cardiology units: data from a large nationwide cardiology database (IN-CHF registry). Am Heart J.

[23] Steinman MA, Patil S, Kamat P, Peterson C, Knight SJ (2010). A taxonomy of reasons for not prescribing guideline-recommended medications for patients with heart failure. Am J Geriatr Pharmacother.

[24] Shang X, Lu R, Liu M, Xiao S, Dong N (2017). Heart rate and outcomes in patients with heart failure with preserved ejection fraction: a dose-response meta-analysis. Medicine (Baltimore).

[25] Pocock SJ, Wang D, Pfeffer MA, Yusuf S, McMurray JJV, Swedberg KB (2006). Predictors of mortality and morbidity in patients with chronic heart failure. Eur Heart J.

[26] McAlister FA, Wiebe N, Ezekowitz JA, Leung AA, Armstrong PW (2009). Meta-analysis: beta-blocker dose, heart rate reduction, and death in patients with heart failure. Ann Intern Med.

[27] Bohm M, Swedberg K, Komajda M, Borer JS, Ford I, Dubost-Brama A (2010). Heart rate as a risk factor in chronic heart failure (SHIFT): the association between heart rate and outcomes in a randomised placebo-controlled trial. Lancet.

[28] Komajda M, Carson PE, Hetzel S, McKelvie R, McMurray J, Ptaszynska A (2011). Factors associated with outcome in heart failure with preserved ejection fraction: findings from the Irbesartan in heart failure with preserved ejection fraction study (I-PRESERVE). Circ Heart Fail.

[29] DeVore AD, Mi X, Mentz RJ, Fonarow GC, Van Dyke MK, Maya JF (2016). Discharge heart rate and beta-blocker dose in patients hospitalized with heart failure: findings from the OPTIMIZE-HF registry. Am Heart J.

[30] Heckman GA, Shamji AK, Ladha R, Stapleton J, Boscart V, Boxer RS (2018). Heart failure Management in Nursing Homes: a scoping literature review. Can J Cardiol.

[31] Plichart M, Orvoen G, Jourdain P, Quinquis L, Coste J, Escande M (2017). Brain natriuretic peptide usefulness in very elderly dyspnoeic patients: the BED study. Eur J Heart Fail.

