# Supplementary material for: National survey on the management of heart failure in individuals over 80 years of age in French geriatric care units
Source: BMC Geriatr. 2019 Aug 1;19:204. doi: 10.1186/s12877-019-1215-y (PMC6670218; doi:10.1186/s12877-019-1215-y)
Supplement: Supplementary file 1 — Table S1 Characteristics of patients according to ACEI or ARB use. Table S2 Characteristics of patients according to β-blocker use. (DOCX 47 kb) [file 12877_2019_1215_MOESM1_ESM.docx]

Table S1, Characteristics of patients according to ACEI or ARB use.

| General characteristics, M (SD) | No ACEI or ARB | ACEI or ARB | p^1^ | p^2^ |
| --- | --- | --- | --- | --- |
|  | N=848 | N=629 |  |  |
| Heart failure type, % (N) |  |  |  |  |
| Decompensated | 24.1 (204) | 17.8 (112) | 0.004 | 0.005 |
| Stable | 75.9 (641) | 82.2 (516) |  |  |
| Age (years) | 88.5 (5.3) | 87.7 (5.0) | 0.002 | 0.005 |
| Women, % (N) | 70.4 (596) | 67.0 (418) | 0.18 | 0.43 |
| Weight (Kg) | 63.0 (15.2) | 66.8 (15.4) | <.0001 | <.0001 |
| Systolic blood pressure (mmHg) | 125 (19) | 128 (19) | 0.0002 | 0.0002 |
| Diastolic blood pressure (mmHg) | 68.7 (11.4) | 69.0 (11.9) | 0.65 | 0.68 |
| Orthostatic hypotension, % (N) | 18.1 (73) | 19.2 (64) | 0.78 | 0.65 |
| Diabetes mellitus, % (N) | 19.2 (163) | 25.9 (163) | 0.003 | 0.01 |
| Low salt diet, % (N) | 10.4 (88) | 8.92 (56) | 0.39 | 0.19 |
| Creatinine (µmol/L) | 105 (58) | 94.6 (41.0) | 0.0001 | <.0001 |
| eGFR (mL/min) | 32.9 (15.5) | 38.0 (17.0) | <.0001 | <.0001 |
| Hemoglobin (g/dL) | 11.8 (1.7) | 11.9 (1.7) | 0.13 | 0.15 |
| Albumin (g/L) | 32.3 (5.5) | 33.3 (4.8) | 0.002 | 0.002 |
| Malnutrition (albumin < 35g/l) | 66.6 (454) | 60.9 (300) | 0.05 | 0.05 |
| Brain natriuretic peptide (ng/mL) | 10917 (90750) | 972 (2301) | 0.18 | 0.31 |
| NTproBNP (ng/mL) | 91722 (502750) | 9687 (50377) | 0.05 | 0.18 |
| Sodium (mmol/L) | 139 (4) | 139 (4) | 0.10 | 0.07 |
| Potassium (mmol/L) | 4.15 (0.52) | 4.25 (0.49) | 0.0004 | 0.0003 |
| Echocardiography available, % (N) | 52.5 (445) | 53.1 (334) | 0.49 | 0.41 |
| LVEF on cardiac ultrasound, % (N) | 54.0 (13.6) | 51.5 (15.3) | 0.02 | 0.05 |
| Atrial fibrillation on ECG, % (N) | 44.9 (367) | 41.0 (247) | 0.16 | 0.11 |
| Total number of drugs | 8.00 (3.30) | 8.99 (3.19) | <.0001 | <.0001 |
| History of myocardial infarction, % (N) | 25.0 (212) | 33.2 (208) | 0.0008 | 0.002 |
| Peripheral arterial disease, % (N) | 18.0 (152) | 22.7 (143) | 0.03 | 0.03 |
| History of stroke, % (N) | 17.6 (149) | 22.9 (144) | 0.01 | 0.02 |
| Dementia, % (N) | 53.5 (453) | 50.6 (318) | 0.29 | 0.32 |
| COPD, % (N) | 24.9 (211) | 24.2 (152) | 0.80 | 0.43 |
| Depression, % (N) | 33.2 (281) | 35.3 (222) | 0.43 | 0.36 |
| History of falls, % (N) | 32.0 (271) | 33.3 (209) | 0.63 | 0.56 |
| Age-adjusted Charlson score | 8.51 (2.20) | 8.46 (2.22) | 0.66 | 0.71 |

M (SD), mean (standard deviation); % (N), percentage (number); eGFR, estimated glomerular filtration rate calculated with Cockcroft formula; LVEF, left ventricular ejection fraction; COPD, chronic obstructive pulmonary disease

1 ANOVA test or χ^2^; 2 Logistic regression adjusted for age and sex

Table S2, Characteristics of patients according to β-blocker use.

| General characteristics, M (SD) | No β-blocker | β-blocker | p^1^ | p^2^ |
| --- | --- | --- | --- | --- |
|  | N=768 | N=709 |  |  |
| Heart failure type, % (N) |  |  |  |  |
| Decompensated | 21.3 (163) | 21.7 (153) | 0.89 | 0.71 |
| Stable | 78.7 (604) | 78.3 (553) |  |  |
| Age (years) | 88.5 (5.2) | 87.8 (5.2) | 0.01 | 0.01 |
| Women, % (N) | 68.1 (522) | 69.7 (491) | 0.52 | 0.23 |
| Weight (kg) | 64.8 (15.6) | 64.3 (15.1) | 0.51 | 0.28 |
| Systolic blood pressure (mmHg) | 127 (18) | 126 (20) | 0.27 | 0.19 |
| Diastolic blood pressure (mmHg) | 69.3 (11.5) | 68.5 (11.7) | 0.18 | 0.11 |
| Orthostatic hypotension, % (N) | 17.9 (67) | 19.2 (70) | 0.72 | 0.58 |
| Heart rate (beats per minute) | 74.7 (12.1) | 73.3 (13.3) | 0.05 | 0.04 |
| Diabetes mellitus, % (N) | 20.1 (154) | 24.1 (171) | 0.07 | 0.10 |
| Low salt diet, % (N) | 8.74 (67) | 10.9 (77) | 0.20 | 0.23 |
| Creatinine (µmol/L) | 98.9 (53.3) | 102 (50) | 0.19 | 0.09 |
| eGFR (mL/min) | 35.7 (16.2) | 34.3 (16.4) | £0.10 | £0.04 |
| Hemoglobin (g/dL) | 11.8 (1.7) | 11.8 (1.6) | 0.93 | 0.92 |
| Albumin (g/L) | 32.7 (5.3) | 32.8 (5.1) | 0.67 | 0.59 |
| Malnutrition (albumin < 35g/l) | 65.6 (389) | 62.6 (365) | 0.31 | 0.24 |
| Brain natriuretic peptide (ng/mL) | 5449 (59793) | 7406 (74399) | 0.79 | 0.78 |
| NTproBNP (ng/mL) | 43145 (254185) | 70756 (481339) | 0.50 | 0.47 |
| Sodium (mmol/L) | 139 (4) | 139 (4) | 0.59 | 0.57 |
| Potassium (mmol/L) | 4.20 (0.50) | 4.18 (0.52) | 0.55 | 0.69 |
| Echocardiography available, % (N) | 45.4 (349) | 60.6 (430) | <.0001 | <.0001 |
| LVEF on cardiac ultrasound, % (N) | 54.8 (13.9) | 51.5 (14.6) | 0.003 | 0.002 |
| Atrial fibrillation on ECG, % (N) | 39.9 (293) | 46.8 (321) | 0.01 | 0.01 |
| Total number of drugs | 8.10 (3.41) | 8.78 (3.12) | <.0001 | 0.0002 |
| History of myocardial infarction, % (N) | 21.7 (167) | 35.8 (253) | <.0001 | <.0001 |
| Peripheral arterial disease, % (N) | 18.6 (143) | 21.5 (152) | 0.19 | 0.13 |
| History of stroke, % (N) | 19.8 (152) | 19.7 (140) | 0.99 | 0.88 |
| Dementia, % (N) | 53.1 (407) | 51.3 (364) | 0.54 | 0.44 |
| COPD, % (N) | 27.2 (209) | 21.6 (153) | 0.01 | 0.005 |
| Depression, % (N) | 35.1 (269) | 33.0 (234) | 0.43 | 0.35 |
| History of falls, % (N) | 31.2 (239) | 33.9 (240) | 0.29 | 0.26 |
| Age-adjusted Charlson score | 8.38 (2.19) | 8.61 (2.22) | 0.05 | 0.02 |

M (SD), mean (standard deviation); % (N), percentage (number); eGFR, estimated glomerular filtration rate calculated with Cockcroft formula; LVEF, left ventricular ejection fraction; COPD, chronic obstructive pulmonary disease

1 ANOVA test or χ^2^; 2 Logistic regression adjusted for age and sex.
